# Supplementary material for: Complex‐centric proteome profiling by SEC‐SWATH‐MS
Source: Mol Syst Biol. 2019 Jan 14;15(1):e8438. doi: 10.15252/msb.20188438 (PMC6346213; doi:10.15252/msb.20188438)
Supplement: Supplementary file 8 — Dataset EV7 [file MSB-15-e8438-s008.zip › feature_plots_string/Q9H0A0.pdf]

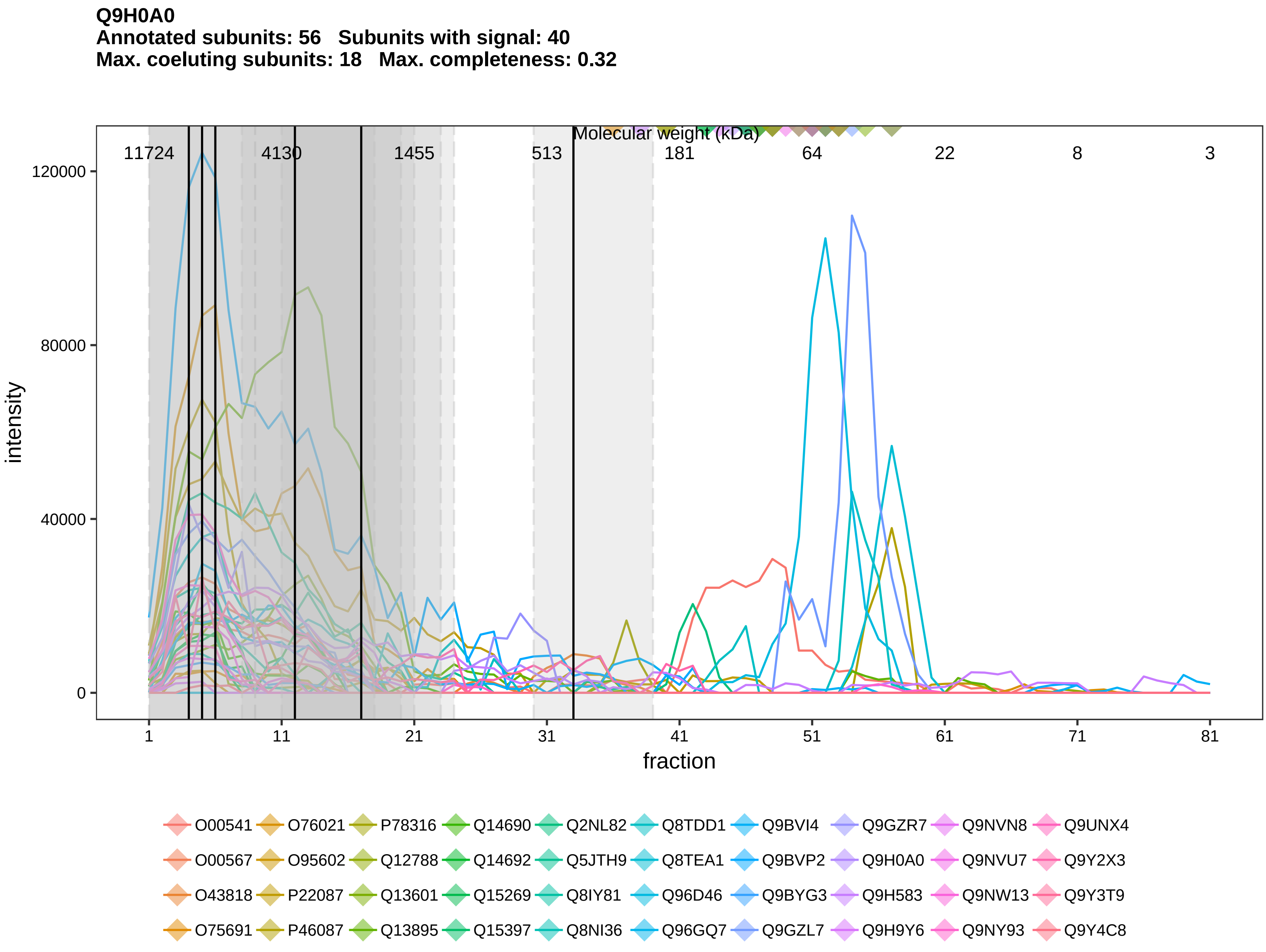
Q9H0A0
Annotated subunits: 56 Subunits with signal: 40
Max. coeluting subunits: 18 Max. completeness: 0.32
Molecular weight (kDa)
11724
4130
1455
513
181
64
22
8
3
intensity
120000
80000
40000
0
fraction
1
11
21
31
41
51
61
71
81
O00541
O76021
P78316
Q14690
Q2NL82
Q8TDD1
Q9BVI4
Q9GZR7
Q9NVN8
Q9UNX4
O00567
O95602
Q12788
Q14692
Q5JTH9
Q8TEA1
Q9BVP2
Q9H0A0
Q9NVU7
Q9Y2X3
O43818
P22087
Q13601
Q15269
Q8IY81
Q96D46
Q9BYG3
Q9H583
Q9NW13
Q9Y3T9
O75691
P46087
Q13895
Q15397
Q8NI36
Q96GQ7
Q9GZL7
Q9H9Y6
Q9NY93
Q9Y4C8
